# The international X-linked hypophosphataemia (XLH) registry (NCT03193476): rationale for and description of an international, observational study

**DOI:** 10.1186/s13023-020-01434-4

**Published:** 2020-06-30

**Authors:** Raja Padidela, Ola Nilsson, Outi Makitie, Signe Beck-Nielsen, Gema Ariceta, Dirk Schnabel, Maria Luisa Brandi, Annemieke Boot, Elena Levtchenko, Michael Smyth, Ravi Jandhyala, Zulf Mughal

**Affiliations:** 1 Royal Manchester Children’s Hospital and Faculty of Biology, Medicine and Health, University of Manchester, Manchester, UK; 2 Karolinska Institutet, Stockholm, Sweden; 3 Örebro University, Örebro, Sweden; 4 Children’s Hospital, Pediatric Research Center, University of Helsinki and Helsinki University Hospital, Helsinki, Finland; 5 Centre for Rare Diseases, Aarhus University Hospital, Aarhus, Denmark; 6 Hospital Vall d’Hebron, Universitat Autonoma Barcelona, Barcelona, Spain; 7 Center for Chronic Sick Children, Pediatric Endocrinology, Charité, University Medicine Berlin, Berlin, Germany; 8 University of Florence, Florence, Italy; 9 University of Groningen, Groningen, The Netherlands; 10 University Hospitals Leuven, KULeuven, Leuven, Belgium; 11 Kyowa Kirin International, Galashiels, UK; 12 Medialis Ltd, Banbury, Oxford, UK

**Keywords:** X-linked hypophosphataemia (XLH), Rare disease, Patient registry, Vitamin D, Disease history, Post-authorisation safety, Bburosumab, Real-world evidence, Quality of life, XLH management

## Abstract

**Background:**

X-linked hypophosphataemia (XLH) is a rare, hereditary, progressive and lifelong phosphate wasting disorder characterised by pathological elevations in fibroblast growth factor (FGF) 23 concentration and activity; XLH has an incidence of approximately 1 in 20–25,000 individuals. Excess FGF23 activity leads to increased phosphate excretion in the kidneys – mediated by downregulation of renal tubular phosphate transporters – and reduced phosphate absorption in the intestines – due to impaired vitamin D activation. This results in impaired bone growth and mineralisation, short and disproportionate stature, leg bowing, musculoskeletal pain, spontaneous dental abscesses, rickets, and osteomalacia. The spectrum of manifestations differs between paediatric and adult patients. Those involved in the treatment of this condition face many challenges, including a lack of robust natural history and demographic data. This multicentre, international, rare-disease patient registry (XLH Registry) was established to address the paucity of data in XLH and to help inform future clinical practice.

**Results:**

The XLH Registry collects standard diagnostic and monitoring practice data, including (where applicable) diagnosis and disease progression history, treatment regimens and family history; the protocol does not mandate any interventions or clinical assessments. The XLH Registry aims to recruit 1200 paediatric and adult patients with XLH over 10 years, and several data analyses and peer-reviewed publications are expected to be generated throughout this period. A post-authorisation safety study for Bburosumab, for which the registry Sponsor is the marketing authorisation holder, will be nested as a sub-study within the XLH Registry via a subsequent protocol amendment.

**Conclusion:**

The data collected within this rare-disease patient registry will be utilised to synthesise real-world evidence to inform the management of XLH, to improve the quality of life and standard of care of patients living with this rare debilitating disease.

## Introduction

X-linked hypophosphataemia (XLH) is a rare, hereditary, progressive and lifelong phosphate wasting disorder characterised by pathological elevations in the serum concentrations and activity of fibroblast growth factor (FGF) 23. This increase in FGF23 is caused by inactivating mutations in the *phosphate regulating endopeptidase homolog, X-linked* (*PHEX*) gene [1]. The exact mechanisms linking *PHEX* and FGF23 remain to be fully elucidated. However, *PHEX* mutations are thought to affect expression, rather than degradation of FGF23 [2–4]. Increased FGF23 activity is responsible for downregulation of the sodium-dependent phosphate co-transporters NPT2a and NPT2c in the proximal renal tubules, alongside diminished synthesis and increased catabolism of active vitamin D due to decreased 1α-hydroxylase and increased 24-hydroxylase enzyme activity. The combination of these physiological changes results in increased phosphate wasting via the kidneys and reduced phosphate absorption in the intestines; these impairments in phosphate homeostasis lead to chronic hypophosphataemia [1]. Chronic hypophosphataemia is responsible for both defective bone mineralisation and tooth formation, leading to the typical skeletal and extra-skeletal manifestations of XLH [1]. XLH affects around 1 in 20–25,000 individuals, meeting the European Union (EU) definition of a rare-disease (affects < 1 in 2000 individuals) [5–8].

Patients affected by XLH display a diverse range of signs, symptoms and diagnoses, which differ not only between paediatric and adult patients but also between affected individuals. Circulating phosphate is integral for optimal growth and mineralisation of newly formed bones; paediatric patients are particularly affected as these impairments in bone growth, and mineralisation are often compounded during periods of rapid growth, such as seen during childhood and adolescence. Children with XLH typically present with symptoms such as progressive bowing of weight-bearing extremities, delayed motor development, impaired and disproportional growth, rickets, and future dental complications. Adults with XLH often display symptoms and signs of osteomalacia such as excessive pain, pseudofractures and osteoarthritis, alongside hearing deficits, increasing numbers of dental complications and abscesses over time, musculoskeletal deficiencies such as stiffness, impaired mobility, weakness and fatigue [9–11]. Many XLH complications seen in adult patients are thought to stem from sub-optimal disease management during childhood [9–11]. All patients, regardless of age, tend to highlight that their condition has a severe negative impact on their quality of life [1, 10, 12]. Evidence from a recent study by Skrinar et al. assessed the lifelong effects of XLH on patients, finding that many symptoms of XLH which emerge in the paediatric period remain unresolved, and continue to impact patients during adulthood [11]. Both paediatric and adult patients, in the study by Skrinar et al., reported substantially worse quality of life than the general population [11]. Hence, findings from our study highlight the need for a greater understanding of the natural history and disease progression profiles of patients with XLH in order to improve the clinical management of the condition.

Initially, the standard of care for patients with XLH was the administration of very high doses of vitamin D; the adjuvant use of phosphate salts was subsequently published in 1964 [13, 14]. Today’s conventional treatment of XLH – popularised in the late 1970s – involves the administration of a combination of oral phosphate and active vitamin D analogue(s) [9, 10], accompanied in some cases by the use of adjuvant therapeutic options such as growth hormone [9]. None of these treatments have undergone regulatory scrutiny and/or approval, suggesting there is no agreed posology, leading to differences between treatment regimens at national – and even regional – levels; an extensive consensus document on the diagnosis and management of XLH has recently been published which aims to address these issues [9]. While these therapeutic regimens have demonstrated improvements in the clinical manifestations of many patients, they only target phosphate and active vitamin D deficiencies and not the disease-causing excess of FGF23 [9, 10]. Conventional therapy may also lead to iatrogenic complications, such as tertiary hyperparathyroidism, which may require additional therapeutic interventions, such as parathyroidectomy or cinacalcet therapy [15]. Further, there has, until recently, been a lack of consensus on the value of conventional treatment in adult patients. Newer guidelines recommend treatment only of symptomatic adult patients, i.e. those with musculoskeletal pain, pseudofractures, dental issues, planned orthopaedic or dental surgery, or biochemical evidence of osteomalacia; the value of treatment in non-symptomatic adults with XLH remains unclear [9, 10].

Recently, a biological treatment – the fully human IgG1 monoclonal antibody (mAb) against FGF23, burosumab – has received regulatory approval in Europe (paediatric and adolescent patients only), the Middle-East, South America, Canada and the USA for the treatment of XLH [16, 17]. Existing evidence suggests burosumab may be superior to conventional therapy, based on findings from a Phase III clinical study in children with confirmed rickets that have not responded adequately to conventional therapy, with similar results observed in a Phase III placebo-controlled study among symptomatic adults burosumab [18, 19]. However, there currently remains a dearth of data surrounding the long-term efficacy, safety and drug usage information for burosumab in the broader ‘real-world’ population.

Furthermore, as with many other rare diseases, there is a limited availability of large-scale data reporting on the epidemiology of XLH, particularly in terms of incidence, prevalence, risk factors, co-morbidity, treatment modalities and mortality on an international level. One of the most effective methods of collecting all of the required large-scale patient data – which has been effectively used in other rare diseases – is the use of registries [20, 21].

Patient registries or disease registries, can either be prospective, retrospective or a combination of prospective and retrospective observational cohort clinical studies, as no specific treatment, clinical assessment or data collection are introduced or mandated as part of the protocol [22]. Though patient registries can collect data on particular treatments, they are exempt from the European Commission’s (EC) Clinical Trials Regulation as the protocol does not mandate any treatment or diagnostic and monitoring practices outside of standard clinical practice [23]. The benefits of this choice of clinical study are beginning to be realised – particularly in rare diseases – not just by those involved in disease management but also by regulatory bodies such as the European Medicines Agency (EMA) [24–27]. Patient registries allow for long-term assessment of patients with much fewer exclusion criteria than typical clinical studies and provide a wealth of disease-specific information.

The XLH patient registry is an international, multicentre, non-interventional, observational cohort clinical study of both paediatric and adult patients with XLH, collecting data retrospectively at study entry and prospectively during follow up. Expert centres for the treatment of XLH across Europe and the Middle East were invited to participate in the XLH Registry and the data to be collected is intended to aid healthcare providers in the optimisation of clinical decision making, through an enhanced understanding of the variability, progression and natural history of XLH, alongside the actual burden of disease.

## Methods

### Registry identification

This rare-disease patient registry has been registered with clinicaltrials.gov, under the identifier NCT03193476.

### Study population

To be eligible for inclusion in the XLH Registry, patients must meet all of the following criteria:

1. Male or female subjects of all ages at baseline.
2. Diagnosis of XLH with clinical, radiological, biochemical and/or genetic findings consistent with XLH.

Any patient meeting any of the following exclusion criteria at baseline cannot be included in the XLH Registry:

1. Patient or their legally designated representative does not have the cognitive capacity to provide informed consent.
2. Patients who are currently participating in an interventional clinical trial.
  - Patients may be approached for inclusion in the XLH Registry once their involvement in the clinical trial (including all trial follow up assessments) has been completed.
  - Participating in a Compassionate Use Program, Pre-commercial Program (i.e. Named Patient Sales, Nominative Temporary Use Authorisation (TUA)) or Investigator-Initiated Study does not preclude a patient from participation in the XLH Registry.

Individuals who are treatment naïve, treated with conventional therapy alone or in combination with adjuvant therapies such as growth hormone or cinacalcet, treated with burosumab, and those with prior XLH targeted treatment histories but not currently receiving treatment are all eligible for inclusion in the XLH Registry.

### Study design

The XLH Registry is an international, multicentre, non-interventional clinical study. It captures treatment details and clinical outcome variables in patients with XLH and patients are followed for as long as informed consent (and assent, where applicable) and regulatory permissions are maintained. Only data collected during standard routine examinations are recorded within the XLH Registry, and no specific examinations/data entries are mandated. Any drug therapy considered necessary for the patients’ welfare may be administered at the discretion of the treating physician; all such treatment and any changes that occur throughout participation in the XLH Registry are recorded in the database.

The XLH Registry was initiated in August 2017, gained its first national regulatory approval in September 2017 (United Kingdom) and the first patient was enrolled on the October 42,017. The XLH Registry will run for 10 years in the first instance, at which point the Sponsor may decide to continue or discontinue the registry, in agreement with the applicable regulatory authorities.

### Registry objectives

The primary objective of the XLH Registry is to collect data to characterise (where applicable) the treatment, burden of disease, disease progression and long-term outcomes of XLH in both paediatric and adult patients and assess the safety of medications used to manage the symptoms and signs of XLH in both paediatric and adult patients.

### Determination of sample size

As this is a prospective, observational register of patients with XLH, there is no specific sample size based on statistical considerations. A survey conducted with XLH clinical experts across Europe provided evidence suggesting a total recruitment target of 1200 eligible XLH patients would be appropriate to enable robust research to be conducted on the data contained within the Registry.

### Ethics

The XLH Registry is run following the recommendations from the Declaration of Helsinki and has received ethical, regulatory and institutional approvals at national, regional and site level for each participating country, as required. Patient data held within the registry is kept in accordance with the EU General Data Protection Regulations (GDPR) on the processing of personal data and the protection of privacy in the electronic communication sector (2016/679/EU).

All eligible patients with XLH visiting a participating clinic are provided with the locally approved patient or parental/caregiver information sheet to review. Age appropriate information sheets have been developed for those patients under the age of 18 (or 16) depending on local guidelines.

Once a patient or their legal representative have agreed to participate and provided informed consent, they are enrolled in the XLH Registry; minors aged ≥12 years are also required to provide their assent before study inclusion. Patient data is link-anonymised, with physicians at the enrolling site retaining an enrolment log to allow patient identification. No pre-determined follow-up requirements apply for the XLH Registry. However, physicians should update the registry promptly after a patient’s visit, once new information is available or, at a minimum, on an annual basis.

Participating centres were reimbursed for associated registry activities including obtaining informed consent, data entry and maintenance of regulatory folders.

### Data collection

#### Determination of data and variables to collect

A systematic assessment of all clinical trials involving patients with a diagnosis of XLH was performed to identify the clinical variables which should be included within the registry. This initial list was then reviewed by members of the steering committee, sponsoring organisation, and service delivery team to determine whether the identified variables should be included, formed part of the standard diagnostic and monitoring practice at participating centres and if, in their opinion, there were any items which were missing and should be included as part of the registry dataset. Following this appraisal, the adjusted list was agreed upon by all members of the steering committee and formed the finalised list of registry data items.

#### Data to be collected

When patients are enrolled into the XLH Registry, both retrospective data [past medical history] and data available at the time of consent [baseline visit] are collected. Prospective data collected post-baseline at all routine clinical visits will be added periodically (at least annually) to the database. As no investigations or data capture outside of routine clinical practice are mandated, only information which is available to the treating physician within this framework is recorded within the XLH Registry. The treating physician will also be responsible for collecting information about compliance with previous XLH medications and drug history. The full list of agreed potential data fields contained within the XLH Registry is presented in Table 1, alongside a summary of the time-points at which specific data is collected (Table 2).

**Table 1:** All data fields which are to be recorded (if collected) for each patient enrolled into the XLH patient registry. Data is grouped under headings, followed by a listing of all individual data variables collected

Variable Group Heading	Variables														
**Informed Consent**	Date	Type	Assent												
**Demographics**	Date of Birth	Biological Gender	Ethnicity												
**XLH-Specific Medication (All XLH-specific medications, including pain medication)**	Dose	Compliance	Duration of Treatment	Reason for Discontinuation											
**Drug History**	Dose	Compliance	Duration of Treatment	Reason for Discontinuation											
**Radiography and Imaging**	Any radiological assessment of disease severity	Type of assessment	Scanner type	Analysis software used											
**Physical examination**	Age	Disease-specific examination													
**Vital Signs**	Temperature	Blood Pressure (Sitting)	Pulse Rate	Respiratory Rate											
**Growth Assessments**	Standing Height (metres)	Sitting Height (metres)	Arm Length (metres)	Leg Length (metres)	Weight (Kg)	Body Mass Index (BMI)	Z-scores (based on national reference)								
**Biochemistry**	1,25(OH)2D	25(OH)2D	Alanine aminotransferase (ALT)	Aspartate aminotransferase (AST)	Amylase	Bilirubin (direct and total)	Blood urea nitrogen (BUN)	Calcium (total)	Chloride	Carbon dioxide (CO2)	Cholesterol (total)	Creatinine	Gamma-glutamyl transpeptidase (GGT)	FGF23	Uric acid
**Biochemistry (continued)**	Intact parathyroid hormone (iPTH	Lactate dehydrogenase	Phosphorus	Potassium	Protein (albumin and total)	Sodium									
**Haematology**	Haematocrit	Haemoglobin	Platelet count	Red blood cell (RBC) count	Mean corpuscular volume (MCV)	Mean Cell Haematocrit (MCH)									
**Urine**	pH	Specific gravity	Protein	Glucose	Calcium	Calcium/creatinine ratio	Phosphorus	Phosphorus/creatinine ratio	TmP/GFR	TRP	Pregnancy test (if applicable)				
**Physiotherapy Reports**	Number of Visits (since last visit)	Use of a Wheelchair	Use of Walking Aids	Use of Medical Devices	Home Adaptations										
**Echocardiogram Reports**															
**Electrocardiogram Reports**															
**Audiology Assessment**															
**Renal Ultrasound Scan**															
**Assessment Tools/Outcome Measure Reports**	Six Minute Walk Test (6MWT)	Timed Up and Go Test (TUG)	Bruininks-Oseretsky Test of Motor Proficiency Section Edition (BOT-2)	Dynamometry											
**Patient Quality of Life Questionnaires or Assessment Reports (May include the following, but this is not an exclusive list)**	Patient-Reported Outcomes Measurement Information System (PROMIS) [for children ≥5 years of age]	Short Form 10 (SF-10) [for children ≥5 years of age]	Pain: Faces Pain Scale-Revised (FPS-R) [for children ≥5 years of age]	Brief Pain Inventory - Short Form [for adult subjects]	Brief Fatigue Inventory - Short Form [for adult subjects]	Short Form 36 (SF-36) [for adult subjects)	Western Ontario and McMaster Universities Osteoarthritis Index (WOMAC) [for adult subjects]	Abbreviated XLH Resource Utilisation Survey	Five-level version of the EuroQoL five-dimensional descriptive system (EQ-5D 5 L) [for adult subjects]	EQ-5D 5 L Proxy [for children < 5 years of age]	Paediatric Musculoskeletal Functional Health Questionnaire (PODCI-POSNA)	General Function Score (GFS)	Health Assessment Questionnaire (HAQ)	Patient Index Data 3 (RAPID3)	Patient Pain Diary
**Social History**	Number of Work/School Dates Missed due to XLH-related Illness since last visit.														
**XLH-Specific Medical, Surgical and Dental History**	Age of Onset of Symptoms	Age at Diagnosis	Diagnosis Method(s)	PHEX Mutation	Family History	Changes to XLH-specific history									
**General Medical History**	Pregnancy and Foetal Outcomes	Gestational/Foetal Exposure to Sponsor Product	Incidence of Hospitalisations	Duration of Hospitalisations	Cause of Hospitalisation	Date of Death	Cause of Death

**Table 2:** Schedule of Assessments for Data Recording. Assessments in bold and italics are mandatory. All others are to be recorded on an if completed basis

Assessments	Study Visit		
	***Retrospective***	***Baseline***	***Prospective***
Site Characteristics		X	
***Informed Consent (and/or assent, as applicable)***^***a***^		X	X^a^
***Demographic information***		X	
XLH-specific Physical Exam		X	
Medical history	X		X
PHEX mutation^b^	X		X^b^
XLH medications	X	X	X
Concomitant Medications		X	
Radiographs and imaging	X	X	X
Physical examination (including dental and audiological assessments)	X	X	X
Vital signs (e.g. heart rate, blood pressure)	X	X	X
Growth assessments (e.g. Height, weight, head circumference)	X	X	X
Physiotherapy	X	X	X
Echocardiogram	X	X	X
Electrocardiogram	X	X	X
Audiology	X	X	X
Renal ultrasound	X	X	X
Patient assessment tools/outcome measures	X	X	X
Exercise Tolerance Tests		X	
Patient Quality of Life questionnaires^c^	X	X	X
Social Impact History	X	X	X

^a^Re-consent to adult registry consent when patient transitions from paediatric to adult patient

^b^PHEX mutation to be recorded during prospective visit if not available during retrospective visit

^c^Quality of Life questionnaires used are dependent upon local clinical practices

#### Data collection tools and data management

Registry data is collected in a purposely designed electronic case record form (eCRF) and data entered into the registry is checked automatically using logical checks (i.e. limits set within the database program). As the XLH Registry does not mandate any investigations or assessments, participating centres can include ‘not done’ information to prevent missing data queries; this function also serves as an assessment of standard of care regimes across centres. If any confounding or missing data are detected, an edit report is generated; the edit reports are sent electronically to the sites for clarification.

Clinical trial data collected in studies conducted by the Sponsor or affiliates will be used for data analysis alongside the registry data, if the informed consent in these studies allows for the data to be used for further research purposes. The addition of previously collected patient data will strengthen the ability of the registry to meet its primary objective.

### Statistical methods

All patients enrolled in the registry are included in the data analysis set. The Medical Dictionary for Regulatory Activities (MDRA) will be used to code medical history, and concomitant disease within the registry and registry internal reports will incorporate information up to and including the latest registry update for each patient. For regular reports on the registry data, all continuous variables are described using standard statistical measures (i.e. number of observations, mean, standard deviation, median, minimum and maximum). All categorical data is summarised in frequency tables.

Patient disposition, demographic data and other baseline characteristics are all treated as described above, while laboratory measurements and other clinical data are tabulated. Medications and medical histories are coded according to the World Health Organisation Drug Dictionary, before being summarised and tabulated. Patient outcome measures and quality of life questionnaires will be summarised and listed.

### Data quality assurance

#### Monitoring and auditing procedures

Registry sites are remotely monitored, and essential registry and site documents are requested for both quality control and storage in the registry master file. Registry sites are regularly contacted via telephone to assist with registry activities and through remote monitoring for queries.

### Electronic Case record forms (eCRF)

The XLH Registry eCRF is completed and signed electronically for each included patient and is completed only by individuals qualified and trained in the completion and data verification of the eCRF information. The physician should ensure the accuracy, completeness, legibility, and timeliness of the data reported in the eCRF. All data requested must be recorded, and any missing data must be explained. Any corrections will overwrite the previous, initial information and an audit trail allowing identification of any modifications will be maintained. Additional requests for confirming or modifying questioned data may be generated through the eCRF, and the investigator will be obliged to respond.

### Management and reporting of adverse events/adverse reactions

Details of adverse event reports associated with any treatment for XLH submitted for an individual patient in the previous year will be captured in the annual registry entry for that patient. Adverse events will be coded with the Medical Dictionary for Regulatory Activities system and described by organ class. Cumulative adverse event information will be reported in the registry’s interim and final analyses.

### Scientific steering committee

An international scientific committee consisting of experts on XLH has been established. This committee consists of Sponsor representatives, European XLH physicians and experts in rare metabolic and endocrine disorders, and European experts in patient registries. Governance principles have been established describing committee members’ responsibilities and obligations, as well as the scientific oversight of the registry’s publication policy; further information adapted from the full registry access agreement can be found below.

Physicians entering data into the registry will have control of their centre’s aggregated data set, and both they and their patients’ will be free to withdraw their consent for their data to be used in analyses at any time; ownership of evidence generated from the aggregated data set will belong to the Sponsor. Access to anonymised data held within the registry is available to all research groups following a successful application to the XLH Registry Steering Committee. The following three steps must be completed as part of the request before access to the anonymised data is provided:

1. Submission of a current access form, providing any applicable supporting documentation,
2. Review of submitted access form and supporting documentation by the XLH Registry Steering Committee (six-week time limit),
3. Following a favourable outcome, applicants will receive a signed letter from the XLH Registry Steering Committee chair, alongside access to anonymised registry data.

The results of all analyses approved by the Steering Committee will be published by the responsible researchers named on the successful access form, with acknowledgement of the Sponsor and contributing investigator sites.

Registry reports are due to be written according to the main objectives of the registry and will be authored in collaboration with the XLH Registry Steering Committee; all statistical analyses performed within the study reports must also be conducted under the supervision of the Steering Committee.

## Discussion

Within the field of rare diseases, patient registries can be an invaluable source of natural history and epidemiological, clinical and medical information which may otherwise be unobtainable by traditional means [28]. Single centre – and even single country-level studies of XLH are unlikely to be able to recruit a sufficient number of patients to allow for statistically robust conclusions to be drawn from the collected data [8]. The implementation and execution of a large, international registry-based study may overcome the potential limitations of smaller individual studies [29].

An area of continuing uncertainty in the management of XLH is the appropriateness of treatment of adult patients. While a recent consensus publication has provided some clarification on current treatment recommendations in the adult XLH patient population, treatment is recommended only in symptomatic adult patients. A large-scale, long-term, patient registry collecting treatment and outcomes data on adults may provide additional evidence that can be used to further refine treatment approaches in this patient group [9]. Registry data may also provide an invaluable source of data on adolescent patients transitioning into adult care; this population is often mismanaged or lost to follow-up care in many diseases, not just in XLH [30, 31].

Furthermore, a multi-national XLH patient registry can collect real-world data not only on natural history and patient demographics, but also on the effectiveness and safety profiles of both conventional therapy and burosumab. The collection of data from patients receiving conventional treatment or burosumab alongside that of patients who are not receiving treatment for XLH may enable the assessment of risk-benefit profiles, informing future clinical practice and improving the quality of care for individuals suffering with XLH.

**Fig. 1:**
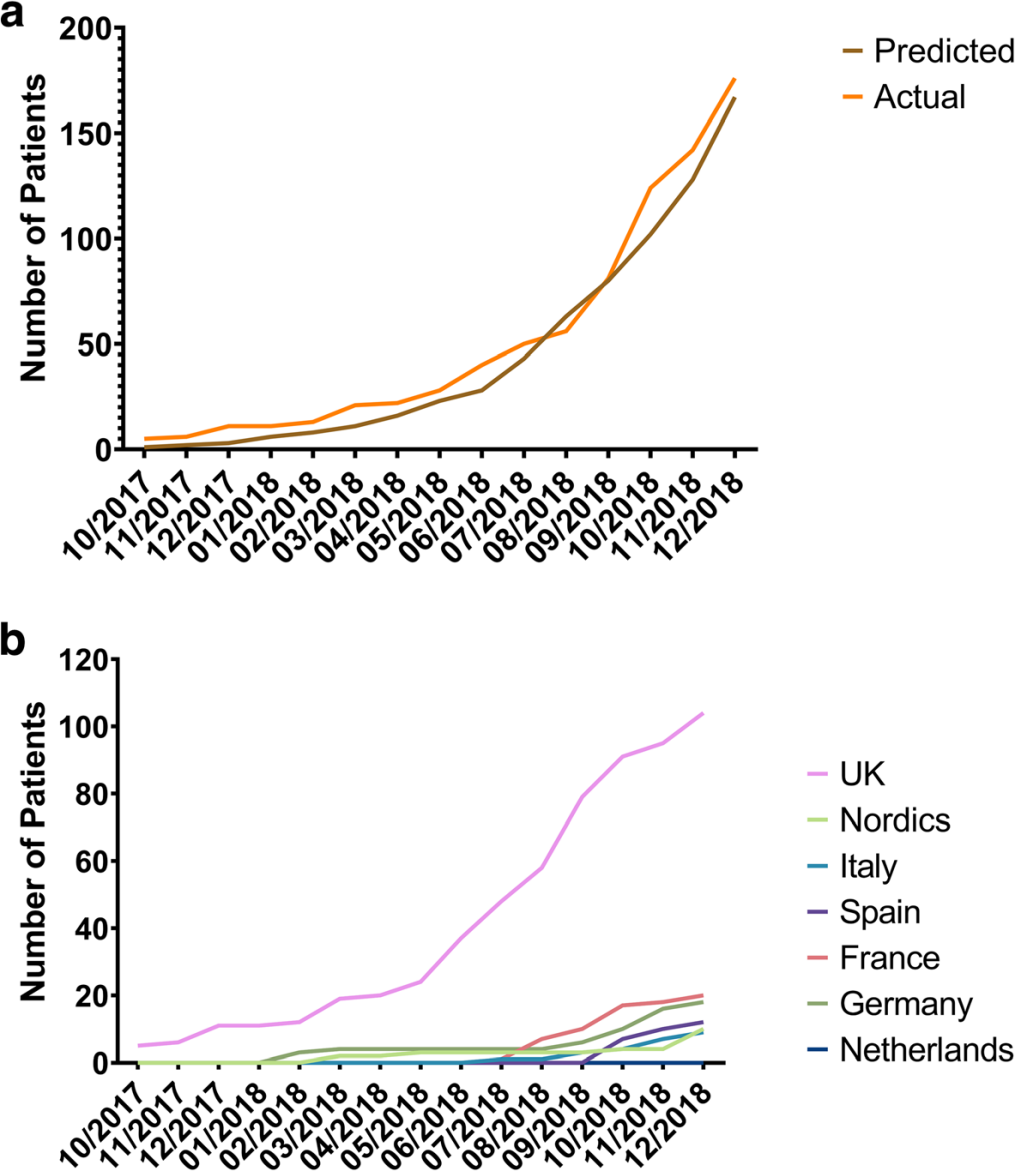
Recruitment curves for the X-Linked Hypophosphatameia Registry. **a** Between October 2017 and December 2018, 176 patients of a target 167 were recruited into the XLH rare-disease registry. **b** Recruitment numbers per country/region

The XHL registry protocol was submitted for ethical approval in all participating countries in August 2017, and the first patient was recruited in October 2017 (Manchester, UK). As of December 2018, 176 patients had been recruited into the registry out of a predicted 167 (105%); this correlates to 15% of the 1200 patient recruitment target (Fig. 1a). A breakdown of recruitment by country and region is shown in Fig. 1b.

Further to the registry protocol described, future work includes the nesting of a Post-Authorisation Safety Study (PASS) for burosumab as a sub-study within the overarching patient registry; regulators typically require a PASS as part of the initial marketing authorisation of a new medicinal product. The flexibility of the registry protocol allows for protocol variations to be quickly processed and implemented, and a full protocol will be published shortly; not all XLH Registry sites are expected to participate in the PASS sub-study as this will require the solicitation of adverse events, which may not be within the capabilities of all participating sites.

### Limitation

A fundamental limitation of this registry study relates to the inconsistency between sites regarding the routine clinical monitoring data they collect. As the study is not a clinical trial, other than informed consent and baseline demographics, there is no mandatory data set which is required to be collected. To mitigate this limitation, work has been conducted with the members of the Registry Steering Committee to generate an XLH-specific Disease Severity Score (DSS) which will form the basis of a Core Dataset. This Core Dataset will help to standardise the information being collected across all sites within the Registry; the DSS will also be freely available to those involved in researching or managing XLH.

Furthermore, we consider the limited applicability of the FAIR (findability, accessibility, interoperability, and reuse of digital assets) principles [32] in its entirety to this clinical study on XLH, a potential limitation. This is due to existing data protection and confidentiality restrictions on the utilisation and sharing of patient-level data. While patient-level data will not be publicly available, evidence generated from aggregated data from the XLH Registry will be publicly available and published in peer-reviewed journals. In addition, as the XLH Registry is registered on ClinicalTrials.gov, with full information accessibility publicly available, suitably qualified individuals can request access to the registry for research purposes. This approach meets the interoperability and reuse components of the FAIR principles [32].

## Conclusion

This observational XLH patient registry aims to recruit 1200 patients with XLH, over 10 years. It has been designed to further the clinical understanding of this chronic, progressive and debilitating genetic disease. It also aims to categorise the demographics, natural histories and treatment histories of both paediatric and adult patients suffering with XLH. The end goal of this registry is the synthesis, publication and dissemination of real-world evidence from the collected data, which can be used to improve both the understanding and clinical management of XLH.

## Data Availability

Not applicable.

## Abbreviations

XLH: X-Linked Hypophosphataemia
FGF: Fibroblast growth factor
PHEX: Phosphate regulating endopeptidase homolog, X-linked
EU: European Union
IgG1 mAb: Immunoglobine G1 monoclonal antibody
EC: European Commission
EMA: European Medicines Agency
PASS: Pre-planned post-authorisation safety study
TUA: Nominative Temporary Use Authorisation
GDPR: General Data Protection Regulations
EQ-5D: Euroqol 5 dimension questionnaire
SF-36: Short Form 36
eCRF: Electronic case record form
DSS: Disease Severity Score
UK: United Kingdom
CRO: Clinical Research Organisation
